# Computational Evidence for Laboratory Diagnostic Pathways: Extracting Predictive Analytes for Myocardial Ischemia from Routine Hospital Data

**DOI:** 10.3390/diagnostics12123148

**Published:** 2022-12-13

**Authors:** Zara Liniger, Benjamin Ellenberger, Alexander Benedikt Leichtle

**Affiliations:** 1 Department of Clinical Chemistry, Inselspital, Bern University Hospital, 3010 Bern, Switzerland; 2 Insel Data Science Center, Inselspital, Bern University Hospital, 3010 Bern, Switzerland; 3 Center for Artificial Intelligence in Medicine (CAIM), University of Bern, 3008 Bern, Switzerland

**Keywords:** laboratory pathways, computational evidence, artificial intelligence, predictive analytes, myocardial ischemia, multiple imputation, orthogonal data augmentation, Bayesian Model Averaging, big data

## Abstract

**Background:** Laboratory parameters are critical parts of many diagnostic pathways, mortality scores, patient follow-ups, and overall patient care, and should therefore have underlying standardized, evidence-based recommendations. Currently, laboratory parameters and their significance are treated differently depending on expert opinions, clinical environment, and varying hospital guidelines. In our study, we aimed to demonstrate the capability of a set of algorithms to identify predictive analytes for a specific diagnosis. As an illustration of our proposed methodology, we examined the analytes associated with myocardial ischemia; it was a well-researched diagnosis and provides a substrate for comparison. We intend to present a toolset that will boost the evolution of evidence-based laboratory diagnostics and, therefore, improve patient care. **Methods:** The data we used consisted of preexisting, anonymized recordings from the emergency ward involving all patient cases with a measured value for troponin T. We used multiple imputation technique, orthogonal data augmentation, and Bayesian Model Averaging to create predictive models for myocardial ischemia. Each model incorporated different analytes as cofactors. In examining these models further, we could then conclude the predictive importance of each analyte in question. **Results:** The used algorithms extracted troponin T as a highly predictive analyte for myocardial ischemia. As this is a known relationship, we saw the predictive importance of troponin T as a proof of concept, suggesting a functioning method. Additionally, we could demonstrate the algorithm’s capabilities to extract known risk factors of myocardial ischemia from the data. **Conclusion:** In this pilot study, we chose an assembly of algorithms to analyze the value of analytes in predicting myocardial ischemia. By providing reliable correlations between the analytes and the diagnosis of myocardial ischemia, we demonstrated the possibilities to create unbiased computational-based guidelines for laboratory diagnostics by using computational power in today’s era of digitalization.

## 1. Introduction

Amongst medical anamnesis, precise clinical examinations, and medical imaging, laboratory parameters play essential parts in diagnostic pathways. They serve as crucial indicators in diagnostic cross-functional flow charts and guide the physician in evaluating different diagnoses. Additionally, they are critical elements in patient follow-up, monitoring therapeutic success, and overall patient care.

It is evident how these laboratory diagnostic qualities contribute to the standards of care [1]. Over the years, expert opinions and publications have determined each parameter’s role; however, opinions vary between clinics, disciplines, and physicians on the relevance of lab parameters in specific diagnostic settings, sometimes neither following guidelines nor evidence-based knowledge [2]. Evaluating diagnostics seems to be done less thoroughly than treatments due to a lack of formal requirements [1]. In short, we require unified, unbiased guidelines regarding the effectiveness of laboratory parameters.

### 1.1. Large but Incomplete Data

In today’s digital world, patient information and laboratory test results are easily accessible. The volume of data for each patient increases with the advancements and diagnostic possibilities in medicine [3]. The recorded data contain a huge amount of yet undiscovered information and relations. A problem that will always occur in large datasets is incompleteness. Due to a certain degree of randomness in request behavior, one will always have missing values in the data matrix. Additionally, it is unlikely to encounter a unified dataset in retrospective studies (as is our case), unlike in prospective studies, where the experimental parameters are standardized beforehand. Previously this has often led to disregarding information and focusing on minimal, but complete data to extract information [4]. The advantage of contemporary computational power is that missing values can be imputed, allowing us to work with larger and still complete datasets.

### 1.2. Human Error and Artificial Intelligence

The human brain seems to face difficulties processing vast amounts of information and tends to reduce the number of inputs when tackling a highly complex situation. This tendency results in solving a problem using a reduced amount of information [5].

The advanced computational power and artificial intelligence (AI) of today can process immense amounts of information and therefore support scientists and physicians in analyzing larger data [3,6]. Artificial intelligence is also capable of learning from mistakes through artificial neural networks. When encountering errors, algorithms can be automatically evaluated and improved [7]. The human mind often lacks such a constant reflection and improvement of approaches, sometimes due to a shortage of time, pride, fixation, etc. [5]. Although machines cannot replace physicians, they have advantages, for example, regarding the following common causes of human error in medicine:

- The unwillingness to question habits, preexisting standards, and diagnoses [5,8]
- The tendency to get stuck on one perspective [5,8]
- The inclination to interpret results to fit one’s beliefs [5,8].

Artificial intelligence can complement human intuition through its information processing and unbiased workways and, therefore, help to reduce human error [7]. Letting computer algorithms analyze data can also help to detect unknown or unthought of correlations between parameters (“hidden variables”), specific diagnoses, and patient outcomes [9].

For instance, in our study, these pattern recognition algorithms empower us to evaluate and complete preexisting diagnostic pathways. Through bypassing expert opinion and prior pathophysiologic knowledge, algorithms can discover new approaches instead of following preexisting paths [1,4,10]. As mentioned by Mayer-Schönberger, this “approach gives credibility to data and less of an emphasis on human intuition […] as human reasoning tends to follow well-known paths rather than breaking out of them” [4].

### 1.3. Our Pilot Study

Considering these possibilities, we designed a pilot study that complements our real-world dataset by recreating missing values through multiple imputations and letting computer algorithms calculate the predictive values for laboratory parameters. The aim is to generate computational-based evidence for specific lab parameter predictive values/effectiveness and, consequently, replace the preexisting algorithms based on expert opinions.

We examined the analytes in diagnostics of ischemic heart diseases because they are a leading cause of emergency hospitalizations and death in our hospital and developed countries in general [11]. In the International Statistical Classification of Diseases (ICD-10), ischemic heart diseases include angina pectoris, acute myocardial infarction, subsequent myocardial infarction, complications following acute myocardial infarction, other acute ischaemic heart diseases, and chronic ischaemic heart disease (coded I20.0 to I25.90) [12]. Underlying these diseases is insufficient coronary perfusion or myocardial oxygen supply due to e.g., atherosclerosis.

The Inselspital (University Hospital of Bern) emergency department provided the data, which were anonymized and did not contain personal identifiers, e.g., names and birth dates. Furthermore, this study was waived by the Cantonal Ethics Committee (dispensation granted No 2014/Z023).

Choosing the diagnosis of myocardial ischemia also enabled us to include many patient cases (in total n = 207,874) and ensure a substantial quantity. Furthermore, these diagnoses have well-evaluated diagnostic guidelines, for example, from the European Society of Cardiology (ESC) [13], which provides an adequate substrate for comparison and proof-of-principle for our approach.

## 2. Materials and Methods

### 2.1. Patient Lab Dataset

We collected patients’ laboratory records from the University Hospital of Bern, including only those with a measured troponin T (TnT) value, which corresponded to about 14% of patient cases. This preselection allowed us to include all the patients with a differential diagnosis of myocardial ischemia or damage. As the crucial element for this diagnosis, TnT would be a part of the lab request of any patient presenting symptoms or risk constellations for myocardial ischemia, unless, in rare cases, further diagnoses are no longer wished for (e.g., palliative care). The diagnosis of myocardial ischemia (ICD-10 code range I20.0–I25.90) was chosen, as mentioned above, due to its frequent presence in the emergency room.

We transformed the patient’s diagnoses from the original representation (primary diagnosis and secondary diagnoses) into a binary indicator determining whether the patient was diagnosed with myocardial ischemia or not. In other words, the patient was either labeled with myocardial ischemia if they had a diagnosis within the respective ICD-10 range or labeled as the control group (any other diagnoses).

Due to this study’s retrospective approach and the individual lab parameters in each patient case, the collected data had missing values. A unified and complete dataset could only have been created if every patient with suspected myocardial ischemia or damage had been tested for the same analytes.

### 2.2. Modelling

The methods described in this section aim to extract predictive lab analytes for myocardial ischemia from the sparse lab measurement matrix. Not all machine learning algorithms can work with incomplete data/a matrix and need to be able to evaluate all rows of a given matrix to come to a conclusion. Bayesian Model Averaging, used in this study, is one such algorithm that can not model *a priori* missingness and requires a dense feature matrix. Therefore, we transformed the sparse matrix into a dense matrix using multiple imputation techniques and generating suitable estimates of the missing measurements. Then, we assessed the analytes’ predictiveness using Orthogonal Data Augmentation/Bayesian Model Averaging with regularization (ODA/BMA).

#### 2.2.1. Multiple Imputation

Multiple imputation is a method to estimate the missing values iteratively. It initially creates a random estimate for a missing value, and then each initially missing value is predicted by regressions based on some or all of the other analytes. Through the continuation of this procedure, increasingly better estimates of each missing value are obtained. The quality of the estimate can be measured by using the Gelman–Rubin convergence statistic [14] across multiple such iterative imputation chains. The Gelman–Rubin convergence statistic measures the variance between and within several chains of the same length. Values closer to 1 indicate that the variance across the differently initialized chains decreased significantly, meaning that the estimate can be considered converged. Notable implementations of this approach involve the mi package [15] for the R language and the mice library [16] for R and the python language.

#### 2.2.2. Orthogonal Data Augmentation/Bayesian Model Averaging with Regularization (ODA/BMA)

To estimate the inclusion probabilities of every analyte into predictive models, we used a data augmentation strategy applying orthogonalization (ODA) as well as the Bayesian Model Averaging (BMA) from the framework developed by Gosh and Clyde et al. [15,17,18]. In our study, however, we had to expose this method to sparse data. Therefore, we tested the ODA/BMA method for robustness by imposing the additional burden of dataset sparsity.

Data orthogonalization helps the sampling strategy used in BMA. The sample-based predictive distribution converges faster to the exact predictive distribution after orthogonalization than if sampling directly from the original model space. BMA additionally creates regression models in a step-wise manner to determine how well the analytes can predict the diagnoses. To avoid computing every possible model, which would lead to $2^{n}$ models, BMA allows us to focus on the most predictable models for further calculations by excluding those that do not perform well. In other words, the algorithms discard models that do not predict myocardial ischemia accurately or involve too many parameters for its prediction. The latter ensures that not only the model’s but also the parameter’s predictive value is augmented. The result is a limited set of models with complementary selectivity, allowing calculations of an analyte’s marginal probability and constituting its overall inclusion probability.

If the inclusion probability of an analyte into a model was 95% and above, we defined this analyte as highly predictive. In conclusion, the higher the inclusion probability of the analyte, the more significant we valued its contribution to the diagnosis of myocardial ischemia.

## 3. Results

In numbers, our data includes n = 207,874 patient cases from 2012 and 2019. 9555 had a diagnosis within the ICD range of myocardial ischemia, leaving 198,319 patient cases as control group.

In total, we could include 1929 different analytes in the study. This large number is due to the heterogeneity of the patient cases other than having myocardial ischemia as a differential diagnosis. Because of their common differential diagnosis, 100% of the cases had a value for Troponin T. To provide a graspable overview, we have sorted the further laboratory analytes according to the percentage (80%, 60%, 40%, and 20%) of the analytes appearing in the patient cases (see Table 1 below). Eight analytes (including Troponin T) were measured in at least 80% of the above-mentioned 207,874 patient cases, 26 in at least 60%, 59 in at least 40%, and 110 in at least 20% of the patient cases.

The resulting data subsets are described in the table below: Table 1.

The results of the algorithms are displayed in the analyte’s inclusion probabilities into models with the most predictive diagnostic value for myocardial ischemia. For each inclusion probability, we calculated a 95% confidence interval based on the different imputation chains used to estimate missing values. For some analytes, no confidence interval was obtained due to the 100% inclusion in all chains.

**Figure 1:**
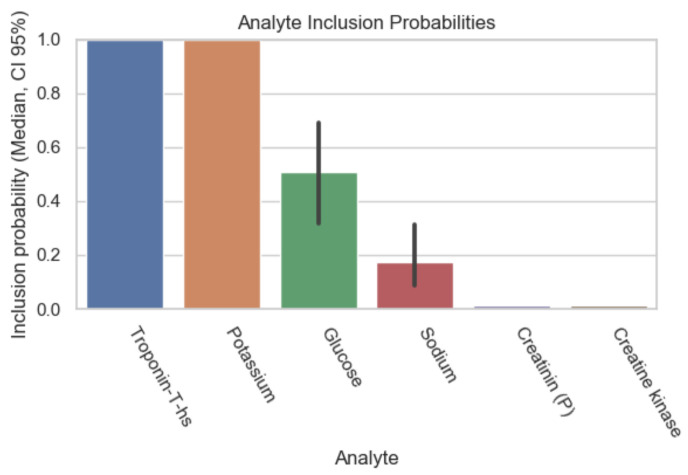
Inclusion probability of parameters based on data subset with initially 20% missing values.

**Figure 2:**
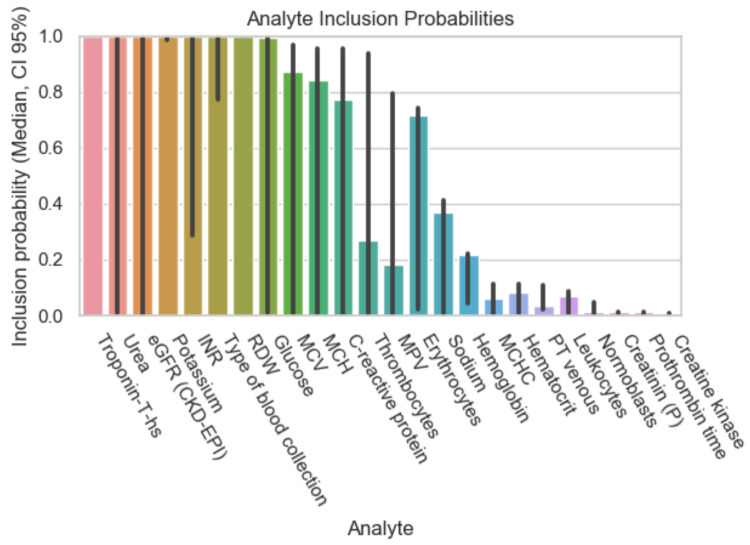
Inclusion probability of parameters based on a data subset with initially 40% missing values.

**Figure 3:**
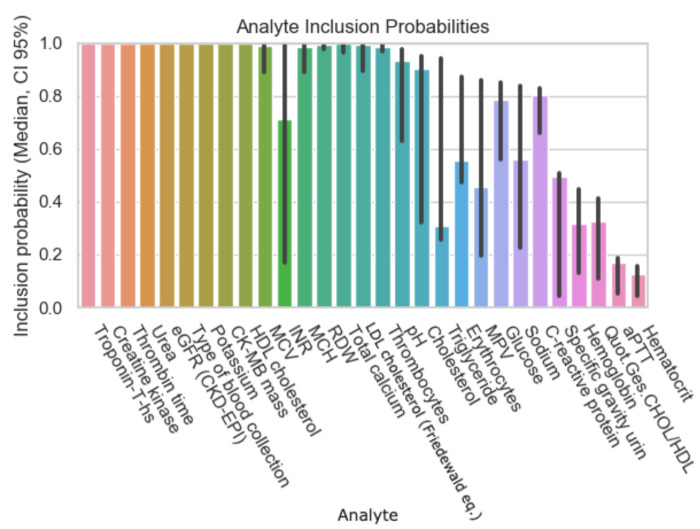
Inclusion probability of parameters based on a subset of the data with (initially) 60% of missing values.

**Figure 4:**
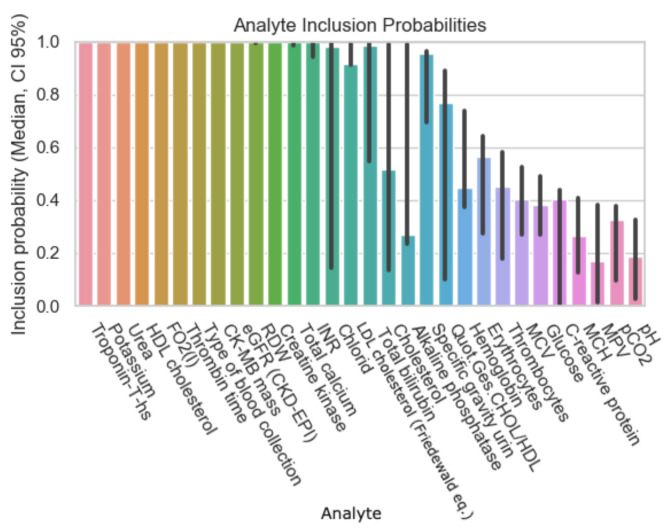
Inclusion probability of analytes based on a data subset with initially 80% missing values.

The following graphs exhibit the calculated inclusion probabilities of each parameter, demonstrating the parameter’s predictive value. As mentioned above, the data were subdivided according to the percentage of missing values in the initial patient records and therefore the results are displayed in Figure 1, Figure 2, Figure 3 and Figure 4.

The bar plot displays the inclusion probability on its vertical axis and the analytes on its horizontal axis. The bars represent the inclusion probability of the analytes with their 95% confidence interval. These inclusion probabilities were calculated with an extract of the data, where not more than 20% of the values were missing. Therefore, only the most commonly tested parameters, when suspecting myocardial ischemia, were included in the calculations. In this case: TnT, potassium, glucose, sodium, creatine kinase, and creatinine. Amongst these, only TnT and potassium showed a high inclusion probability and, therefore, a high predictive value.

More analytes have a high predictive value with a bigger data subset. Those showing an inclusion probability of 95% and above are the following: TnT, potassium, urea, eGFR (glomerular infiltration rate), RDW (red distribution width), glucose, INR (international normalized ratio), and the type of blood collection (arterial or venous).

In comparison to Figure 2, the number of analytes with a 95% and above inclusion probability has doubled in Figure 3, and their confidence intervals have narrowed. In addition to the analytes mentioned in Figure 2, the following have a 95% or more inclusion probability: thrombin time, CK-MB-Mass, CK, HDL (high-density lipoprotein), total calcium, LDL (low-density lipoprotein), MCV (mean corpuscular volume), MCH (mean corpuscular hemoglobin), and MCHC (mean corpuscular hemoglobin concentration). Unlike in Figure 2, “glucose” does not show a high inclusion probability.

The number of analytes with an inclusion probability of 95% and above has not changed significantly, but the types have, as follows: FiO2 (fraction of inspired oxygen), bilirubin, and chloride now show a high inclusion probability, the latter however with a noticeable broad confidence interval. Unlike in Figure 3, MCV, MCH, MCHC, and LDL do not show a 95% or above inclusion probability anymore in Figure 4.

## 4. Discussion

### 4.1. Interpretations of the Results

We found that analytes with a high inclusion probability in the created predictive models have a known association with diagnostic evaluation or the risk factors of myocardial ischemia. TnT, the primary analyte for myocardial ischemia and damage, shows a 100% inclusion probability in all four data subsets (cf. Table 2). This finding was expected, as the diagnosis of myocardial ischemia is currently based nearly exclusively on an abnormal TnT. However, it also indicates that our algorithms effectively extracted the critical analyte for myocardial ischemia and, therefore, served as proof of principle (see below).

Rather than being involved in the diagnosis of myocardial ischemia, most of the parameters show an association with the risk factors, severity, and mortality of coronary heart disease (cf. Table 3). Regarding the different data subsets, we find that the more missing values we allowed in the data subset, the more analytes showed a significant inclusion probability. Provided an accurate data imputation, we can conclude that the bigger the data, the more extracted information. We will discuss further quality improvements of the imputed values below (see Section 4.2).

By looking at the data collection, we can gain an understanding of why the number of analytes increased with the data sparsity. The reason for missing data in this study’s source mentioned above partly lies in the problem addressed in the current diagnostic pathways: although there are existing guidelines, there is no consistent approach to laboratory diagnostics, at least for retrospective approaches. The other contribution to the variation of the analytes lies in the patient’s other differential diagnoses. Therefore, although there may be a joint base, each patient case comes with an individual set of tested analytes. Although this leads to an increased number of missing values, it is an opportunity to examine other tested analytes that we may otherwise not associate with myocardial ischemia.

#### 4.1.1. TnT, CK, CK-MB-Mass

As mentioned above, the fact that TnT is in every predictive model shows that the algorithm recognized the defining parameter for myocardial ischemia. However, it is less obvious that CK (creatine kinase) and CK-MB Mass (creatine kinase myocardial band mass) only show a high inclusion probability in two data subsets. This observation is in contrast to the fact that they are both supposed to be relevant and frequently included in myocardial ischemia diagnostics. The algorithm may have limited their inclusion as they do not define the diagnosis without TnT but rather give us more information regarding the myocardial ischemia size and the timing of myocardial ischemia [13,19]. The algorithm, therefore, cannot gain additional information concerning the diagnosis. This means, concerning myocardial ischemia, CK would only cover the shared variance with troponin. Another possible explanation for its rare inclusion of CK is that its values are elevated in other constellations, e.g., trauma, preceding operations, and physical activities [19], and are not solely specific to myocardial ischemia.

#### 4.1.2. Potassium, eGFR, Urea

Apart from TnT, potassium revealed a high inclusion probability in all models. Rather than defining myocardial ischemia diagnosis, potassium correlates with its underlying diseases and secondary effects.

The maintenance and regulation of potassium levels are up to 80% a result of kidney function. Potassium’s underlying regulatory mechanisms, being such an essential electrolyte in cardiological and neurological functions, are highly robust. Other than different retention parameters (e.g., urea), potassium rises in a stage of severe malfunction of the kidney [20]. So, high potassium levels often reflect severe renal insufficiency/renal failure.

Our algorithms most likely included potassium in numerous models due to the pathophysiologic interaction of renal and cardiac failure and the encapsulated information on common risk factors of coronary heart disease and renal failure.

Renal and cardiac failure have an intricate dependence known as the cardiorenal syndrome, which defines one organ’s resulting failure due to a preceding failure of the other organ, heart, or kidney. An acute decline in heart function (due to, e.g., coronary heart disease or cardiogenic shock) can result in acute kidney injury, known as type 1 acute cardiorenal syndrome [21]. Patients with cardiac failure also show higher mortality when presented with renal failure at the same time [22].

If we look at what causes renal or cardiac failure, it is evident that many common mechanisms/shared risk factors link kidney and heart disease pathologies. For example, diabetes is the most common cause of chronic renal insufficiency due to diabetic nephropathy but also causes diabetic cardiopathy increasing the risk of cardiac failure [23,24].

Considering that potassium already serves as a valuable marker for renal failure, it at first does not seem logical that eGFR (estimated glomerular filtration rate) also shows a high inclusion probability in three out of four figures.

(1) $$\begin{array}{c} GFR=141\times min{\left(S_{cr}/\kappa ,1\right)}^{a}\times max{\left(S_{cr}/\kappa ,1\right)}^{-1.209}\times 0.993^{Age}\\ \times \;1.018(if\;female)\times 1.159(if\;black) \end{array}$$

Equation (1): CKD-EPI equation for the glomerular filtration rate. “*Scr*” is the serum creatinine in mg/dL; gender dependent factor “*k*” equals 0.7 (woman) or 0.9 (man); gender dependent factor “a” equals −0.329 (woman) or −0.411 (man); “*min*” refers to the minimum of Scr/k or 1; “max” refers to the maximum of Scr/k or 1; Age in years [25].

Example for the female Caucasian patient, 53 years of age, with a serum creatinine of 0.8 mg/dL:

(2) $$\begin{array}{c} GFR=141\times min{\left(0.8/0.7,1\right)}^{-0.329}\times max{\left(0.8/0.7,1\right)}^{-1.209}{\times 0.993}^{53}\\ \times \;1.018=88\;\mathrm{mL}/\min/1.73\;m \end{array}$$

However, looking at the formula of the estimated glomerular filtration ratio (CKD-EPI equation), there are additional components included: creatinine, age, race (in the obsolete version), and sex. An increase in creatinine provides an estimate of the renal function and predicts unfavorable outcomes in acute coronary heart syndrome [11]. Age and sex add valuable information to the pre-test probability of myocardial infarction. Male sex and age are known risk factors for coronary heart disease, the latter having an additional strong correlation with adverse outcomes in ACS [11]. Adding eGFR to the models is like adding a whole package of information and risk factors at once.

Urea also shows a high inclusion probability in 3 out of the 4 data subsets. Urea (in some countries expressed as BUN (blood urea nitrogen) [26]) can serve as a supplementary marker for renal insufficiency, but interestingly also seems to correlate with the prognosis of an acute coronary syndrome in mortality risk assessments [11].

#### 4.1.3. INR, Thrombin Time, Bilirubin

International Normalized Ratio (INR), a calculated analyte for the extrinsic coagulation pathway, and the thrombin time were also among those with a high inclusion probability (INR in Figure 2, Figure 3 and Figure 4, thrombin time in Figure 3 and Figure 4). An increased INR value or increased thrombin time points at the use of certain anticoagulants (Marcoumar, Dabigatran, etc.), often due to atrial fibrillation. Including the INR/thrombin time in the predictive models takes patient profiles with underlying heart diseases into account. Another reason the INR could be a predictor for cardiogenic diagnoses may be the influence of congestive heart diseases on liver function. Acute and chronic heart failure can cause a mild elevation of the INR and bilirubin through venous congestion and decreased oxygen supply [27,28]. In this relation, we would expect NT-proBNP to be amongst the predictive analytes, so, surprisingly, the parameter for congestive heart failure itself is not represented in any figures. One could argue that having the marker for secondary complications (as a predictive analyte (here INR) rather than the cause) implies existing complications and a particular magnitude/stage of congestive heart failure.

#### 4.1.4. Type of Blood Collection, FiO2

The type of blood collection includes venous and arterial blood tests. It is not a lab test itself, so we did not expect to be incorporated into the predictive models. Patients who get an arterial blood collection typically either show a reduced general condition—where the physician relies on the fast analysis of the pH, the FiO2, the electrolytes, and glucose—or suffer from dyspnea as their cardinal symptom. In conclusion, patients who get an arterial blood collection are usually in a more critical state. Therefore, the type of blood collection can mirror the physician’s judgment of the patient. In this context, the algorithms could have correlated the type of blood collection and its implied critical patient state to a higher likelihood of myocardial ischemia.

Adding FiO2 to the equation, the models gain the additional knowledge that a blood gas analysis has been conducted.

#### 4.1.5. Red Blood Cell Distribution Width (RDW), MCV, MCH, MCHC

The RDW indicates the heterogeneity of the red blood cells. In the study of Zalawadiya et al., they found a strong correlation between RDW and future cardiovascular events, independent of confounding factors like anemia and renal function. The exact pathophysiological mechanism behind it is uncertain. Chronic subclinical inflammation, high oxidative stress, nutritional deficiencies, and aging are common underlying causes for a broad RDW and the risk for cardiovascular events, which might explain the strong correlation [29].

MCV, MCH, and MCHC show a high inclusion probability (cf. Figure 4). Being markers of anemia could explain the correlation, as severe anemia can cause myocardial ischemia. Interestingly, hemoglobin itself does not display a high inclusion probability. It is unclear what added value MCV, MCHC, and MCH have over hemoglobin, indicating a stronger correlation to myocardial ischemia. Possibly, hemoglobin is more sensitive to preanalytic errors (hemolysis), leading to higher variability in its values and, therefore, lacking essential characteristics for a predictive parameter.

#### 4.1.6. HDL Cholesterol and LDL Cholesterol

Both abnormal HDL and LDL cholesterol are known risk factors for atherosclerosis and, consequently, cardiovascular diseases. In this study, HDL shows a three times higher inclusion probability in predictive models than LDL. That would imply a stronger association of HDL cholesterol with myocardial ischemia than its counterpart. This observation might mirror a stronger negative correlation between abnormal HDL cholesterol and coronary calcification than a positive correlation with LDL cholesterol [30] or also a robust predictive value of HDL cholesterol and the development of a metabolic syndrome [31].

The other way of looking at the results is that the model does not gain significant information by putting both HDL and LDL cholesterol into the same model, considering that dysregulation usually appears simultaneously in various lipoprotein types [32]. Although LDL cholesterol is an established risk factor for atherosclerosis, some studies suggest that Non-HDL cholesterol (as the difference between total cholesterol and HDL cholesterol) captures the cardiovascular risk more thoroughly by including other low-density lipoproteins [33,34]. Therefore, HDL cholesterol unites more information than LDL cholesterol alone. In the current guidelines of the European Society of Cardiology, it is recommended to measure non-HDL cholesterol and apolipoprotein B for cardiovascular risk evaluation instead of LDL cholesterol, as values can be inaccurate, e.g., in patients with diabetes, high triglyceride [35] and analytical issues to measure LDL can occure.

#### 4.1.7. Total Calcium

Calcium is represented as a highly predictive analyte in two out of four models. An abnormal calcium blood level appears yet again to reflect a risk constellation rather than a diagnostic tool. Studies suggest that high levels of calcium or calcium intake over a more extended period can increase the risk for cardiovascular diseases [36,37].

#### 4.1.8. Total Glucose

Following the trend of identifying cardiovascular risk factors, glucose shows a high inclusion probability in one model. We would expect glucose to appear more often in models as an expression of diabetes and a well-known cardiovascular risk factor. Diabetes may nevertheless be represented in analytes other than glucose, possibly, through its long-term effects, such as renal failure due to diabetic nephropathy.

#### 4.1.9. Chloride

Chloride showed a high inclusion probability in 1 out of 4 models. There does not seem to be a known direct association between an abnormal chloride level and myocardial ischemia. However, some studies have found a correlation between mortality in critically ill patients and hyperchloremia. This correlation could be explained, amongst other reasons, by the administration of chloride-rich solutions during resuscitation [38,39]. Changes in chloride levels may also represent the intake of diuretics commonly administered in patients with heart insufficiency, hypertension, etc., therefore, in patients with a higher risk for myocardial ischemia. Additionally, an elevated chloride level can occur in metabolic acidosis due to loss of bicarbonate seen in renal failure. We elaborated above on how renal failure and myocardial ischemia interact.

### 4.2. Strengths and Limitations of the Study

Our approach’s strength lies in overcoming any preceding assumption and analyzing a large clinical dataset. The algorithms do not have and do not need to include prior knowledge concerning predictive analytes for myocardial ischemia, and through earlier multiple imputations, we did not have to exclude patient cases due to missing values. This approach allows us to explore a vast amount of possibilities and correlations for further investigation.

How do we have any evidence that the correlations are not random? As a proof of principle, the algorithms included TnT in all models, proving the algorithms’ capacity to extract the most critical analyte. Again, critics might argue that only patients with a value for TnT were selected for the study introducing a (pre-)selection bias, thereby making it highly suggestible that TnT would be included in all the models. However, it does not automatically imply that the algorithms would make that connection as we did not supply it with any such information.

It would be a misconception to think that these results do not have to be questioned and verified. For example, the analytes with high inclusion probabilities represent previously known risk factors. We can argue that the probability of diagnoses rises with the number of risk factors. However, we cannot dispute that some analytes will not serve as diagnostic parameters, especially in an emergency setting. Furthermore, by choosing myocardial ischemia, we limited the possibility of gaining additional predictive analytes as the diagnosis in the meantime mainly relies on TnT as the only lab parameter. This restriction makes it even more impressive that the algorithms could extract additional analytes that are associated with myocardial ischemia even though they are not of direct diagnostic use.

Although we aimed to select a limited number of highly predictive analytes by implying Bayesian Model Averaging (BMA) with penalization, our results display many analytes with a high inclusion probability. In the future, we would need to improve the algorithm to restrict the number of included analytes, for example, by using a stricter setting of Occam’s window to only include the most predictive analytes. Again, in this study, this restriction would most likely lead to including TnT solely into a predictive model, as myocardial ischemia is in general diagnosed with a rise in TnT blood levels [13]. Future studies with different diagnoses (based not only on mainly one laboratory analyte) can explore suitable algorithms capable of filtering the most predictive set of analytes in cases, where not a single analyte constitutes the diagnosis. A possibility is to add “Ridge and Lasso regularization” [40] to the linear prediction models. We could expect this regularization to induce more sparsity on the number of included features, allowing us to tune the model search toward favoring models with fewer features. It will decrease the inclusion probabilities of less predictive analytes and produce fewer analytes with high inclusion probabilities, resulting in only the most predictive ones under the regularization constraint. The commonly known penalty parameter λ for tuning the regularization can now favor smaller models with the least but most predictive analytes.

A further limitation is that our results lack information on the value of each analyte itself. Our algorithms only extracted the analyte type and not whether its increased or decreased value is associated with the diagnosis. We would need to provide standard values for each analyte to include in the calculations, even at the risk of compromising an unbiased system by having a reference value and adding prior knowledge. Especially in such a setting, it is crucial to have an additional control mechanism for the quality of imputed data to avoid measurement-induced biases [41].

We need to address another challenge that lies in the multiple imputation process concerning missing standard values. During the imputation of missing values, the algorithms assume a specific distribution based on existing values for each analyte. In analytes with a high degree of missingness, the algorithm cannot adequately foretell the distribution; therefore, the distribution of the values tends to resemble one of a Gaussian distribution. Ideally, the algorithm would use the specific distribution of each analyte based on our current knowledge of standard values to impute missing values. Adding standard values would avoid assuming a wrong distribution in analytes with a lot of missingness and correct assumed distribution based on a dataset of ill/symptomatic patients.

Another possibility to improve the data quality is to replace the standard multiple imputation algorithms with an overimputation method, adding additional procedures to ensure an accurate estimate of missing values: overimputation targets not only missing values but also measurement errors in the existing data. The latter is essential to address, as by gaining quality in the observed data, the prior information on the distribution of the values will improve. Overimputation incorporates posterior distributions while generating missing values, making it more efficient than the multiple imputation techniques, where estimates, broadly speaking, are imputed at random and evaluated with an iterative process [41].

Regarding our comprehensive big data approach, we currently lack external verification. This study’s data are sourced from only one hospital and, as mentioned in the introduction, is limited by hospital-specific laboratory diagnostics guidelines and opinions. By including other hospitals as sources, possibly internationally, the data variability would increase significantly. When gathering information from different sources, we would need to ensure the formal and semantic standardization of the data. The challenge would lie in maintaining unified definitions and a common vocabulary [9]. Approaches such as FAIR (findable, accessible, interoperable, reusable) data sharing are being addressed to reduce the barriers concerning data sharing, such as privacy, security, and lacking interoperability. Efforts to standardize data collection by creating data units with a common denominator, so-called common data elements (CDE), are already in place. However, the implementation needs to be extended further [42].

Although more similar work is being presented with the same goal of creating functioning artificial intelligence models in improving diagnosis [43,44], a direct comparison is often limited as the algorithms vary in each project. As mentioned in one of these studies by Eurlings et al. [43], the models are often not compared to each other and are also not applied to other data for validation. A lively exchange between the projects with the aim of optimizing the models is therefore essential. In a review by Wang et al. [45], different approaches have been looked at concerning artificial intelligence in acute coronary syndrome. The mentioned projects using AI in the diagnosis of acute coronary syndrome place their focus on diagnostic tools other than laboratory analytes, such as imaging, or focus on pre-test probability and prediction of necessary management.

## 5. Conclusions

In this study, we demonstrate that our algorithms are capable of extracting predictive analytes for myocardial ischemia without any prior knowledge. Further, we show that a sparse matrix in the original dataset does not have to result in using less but complete data and that algorithms could analyze a large, complemented dataset.

Concretely, we show that using multiple imputation techniques allows us to replace missing values in the original matrix without the apparent introduction of bias to the data. This completed dataset enabled us to apply orthogonal data augmentation (ODA) and Bayesian Model Averaging (BMA) to construct predictive models for our exemplary case of myocardial ischemia. The used ODA/BMA method proved capable of defining the most crucial analyte in diagnosing myocardial ischemia: TnT. Even though the diagnosis we chose limited the number of extractable predictive analytes, the algorithms garnered further valuable information from the data by selecting analytes associated with surrounding risk factors or the mortality of myocardial ischemia.

Choosing the myocardial ischemia in this pilot study allowed us to verify the plausibility of the algorithms as it dwells in a well-defined, researched, and accepted diagnostic framework.

Despite the challenges, the algorithms used in this study provide groundwork we can further build on. If we can overcome the obstacles and limitations mentioned above—by, for example, restricting the model size and therefore selecting only the most predictive analytes, by adding standard values to each analyte to improve the imputation process, and by extending the source to other hospitals—we can strive for the ultimate goal, i,e., to create working algorithms for less explored diagnoses and discover correlating analytes, whether for the diagnosis itself or its risk factors, ultimately improving diagnostics and patient care and replacing opinion-based diagnostics with evidence-based ones.

Importantly, we aim to contribute to assembling algorithms that can cope with the vast, but also highly heterogeneous amount of data recorded in hospitals. We cannot afford to have so much data at our fingertips and not utilize it to improve diagnoses, patient care, and our medical system in general. In this era of artificial intelligence, subsequent studies building on our approach will eventually contribute to making data analyses faster, more in-depth, and as unbiased as possible.

## Figures and Tables

**Table 1:** Description of the created data subsets based on the percentage of sparsity and the resulting number of analytes included.

Dataset ID	Number of Analytes
20% sparsity	8
40% sparsity	26
60% sparsity	59
80% sparsity	110

**Table 2:** Summary of highly predictive analytes. This table summarizes the analytes that show a 95% and above inclusion probability. There are only two analytes that meet these criteria in all four data subsets: TnT and potassium. Most analytes are represented in the data subset with 80% initially missing values, followed by the data subset with 60% initially missing values. Inclusion probablity 95% and above of an analyte in models based on data subsets with initially 20–80% missing values.

Analyte	Inclusion Figure 1	Inclusion Figure 2	Inclusion Figure 3	Inclusion Figure 4	Total Inclusion
Troponin	+	+	+	+	4/4
Potassium	+	+	+	+	4/4
Type of blood collection		+	+	+	3/4
Red Distribution width		+	+	+	3/4
eGFR		+	+	+	3/4
Urea		+	+	+	3/4
INR		+	+	+	3/4
CK			+	+	2/4
CK-MB-Masse			+	+	2/4
Thrombin time			+	+	2/4
HDL-Cholesterol			+	+	2/4
Calcium total			+	+	2/4
FO2				+	1/4
Bilirubin				+	1/4
Chloride				+	1/4
Glucose		+			1/4
LDL-Cholesterol			+		1/4
MCV, MCH, MCHC			+		1/4

**Table 3:** Median and IQR of the analytes with an inclusion probability of 95% and above. Based on Table 2, the values for each analyte are included with an inclusion probability of 95% and above in at least one of the figures listed with the median value and IQR. Columns 2–4 are divided into patients without myocardial ischemia (non-MI), with myocardial ischemia (MI), and the total number (total). In Table 3, the unit of measurement is noted in the first column with the analyte, and the median value and the IQR (placed in the square bracket) are displayed in columns 2–4. As “Type of blood collection” does not come with a value, calculations here were impossible; therefore, its value was placed at zero. Note that FO2 contains data from both, arterial and venous blood.

Analyte Name	NON-MI	MI	Total
Troponin T [ng/L]	20[43.02]	248.1[1233.44]	25[78.62]
Potassium (mmol/L)	4 [0.5]	4.1 [0.5]	4.1 [0.5]
Type of blood collection	0[0]	0[0]	0[0]
RDW (%)	13.5[1.9]	13.4[1.6]	13.5[1.9]
eGFR (mL/min/1.73 m ${}^{2}$)	85[40]	76[36]	84[39]
Urea (mmol/L)	5.9[4.4]	6.3[4.1]	5.9[4.4]
INR	1.1[0.13]	1.03[0.13]	1.01[0.07]
CK (U/L)	96[126]	191[500]	103[154]
CK-MB-Masse (%)	3.9[7.2]	12[44.3]	4.6[11.5]
Thrombin time (s)	16[2.5]	17.6[23.2]	16.1[2.5]
HDL-Cholesterol (mmol/L)	1.24[0.24]	1.14[0.47]	1.23[0.55]
Calcium total (mmol/L)	2.25[0.21]	2.21[0.17]	2.24[0.2]
FO2 (mmHg)	21[41]	59[54]	32[43]
Bilirubin (µmol/L)	8[10]	9[8.5]	8[10]
Chloride (mmol/L)	107[7]	108[5]	107[7]
Glucose (mmol/L)	5.96[2.11]	6.5[2.5]	6[2.1]
LDL-Cholesterol (mmol/L)	2.23[1.36]	2.39[1.49]	2.25[1.38]
MCV (fl)	86[7]	86[6]	86[7]
MCH (pg)	30[3]	30[2]	30[3]
MCHC (g/dL)	342.5[17]	344[17]	343[17]

## Data Availability

Data are available upon request under the conditions of ethics and institutional clearance. The code for this project is available at https://github.com/leichtle/computational-diagnostic-paths (accessed on 2 December 2022).

## Abbreviations

The following abbreviations are used in this manuscript:

AI	artificial intelligence
BMA	Bayesian Model Averaging
CK	creatine kinase myocardial band
CK-MB	creatine kinase MB
DOAJ	Directory of Open-Access Journals
FiO2	fraction of inspired oxygen
GFR	glomerular Filtration Rate
HDL	high-density lipoprotein
ICD	International Statistical Classification of Diseases
INR	international normalized ratio
IQR	interquartile range
LD	linear dichroism
LDL	low-density lipoprotein
MCH	mean corpuscular hemoglobin
MCHC	mean corpuscular hemoglobin concentration
MCV	mean corpuscular volume
MDPI	Multidisciplinary Digital Publishing Institute
MI	myocardial ischemia
Non-MI	patients without myocardial ischemia
NT-proBNP	N-terminal prohormone of brain natriuretic peptide
ODA	Orthogonal Data Augmentation
RDW	red distribution width
TLA	three letter acronym
TnT	troponin T
